# Engineering Photoactivatability in Genetically Encoded Voltage and pH Indicators

**DOI:** 10.3389/fncel.2019.00482

**Published:** 2019-10-29

**Authors:** Sungmoo Lee, Yoon-Kyu Song, Bradley J. Baker

**Affiliations:** ^1^Center for Functional Connectomics, Brain Science Institute, Korea Institute of Science and Technology, Seoul, South Korea; ^2^Program in Nano Science and Technology, Graduate School of Convergence Science and Technology, Seoul National University, Seoul, South Korea; ^3^Advanced Institutes of Convergence Technology, Suwon, South Korea; ^4^Division of Bio-Medical Science and Technology, KIST School, University of Science and Technology (UST), Seoul, South Korea

**Keywords:** voltage indicator, photoactivatable, GEVI, pH sensor, ecliptic pHluorin, PA-GFP, PA-Bongwoori-R3, PA-ecliptic pHluorin

## Abstract

Genetically-encoded indicators of neuronal activity enable the labeling of a genetically defined population of neurons to optically monitor their activities. However, researchers often find difficulties in identifying relevant signals from excessive background fluorescence. A photoactivatable version of a genetically encoded calcium indicator, sPA-GCaMP6f is a good example of circumventing such an obstacle by limiting the fluorescence to a region of interest defined by the user. Here, we apply this strategy to genetically encoded voltage (GEVI) and pH (GEPI) indicators. Three photoactivatable GEVI candidates were considered. The first one used a circularly-permuted fluorescent protein, the second design involved a Förster resonance energy transfer (FRET) pair, and the third approach employed a pH-sensitive variant of GFP, ecliptic pHluorin. The candidate with a variant of ecliptic pHluorin exhibited photoactivation and a voltage-dependent fluorescence change. This effort also yielded a pH-sensitive photoactivatable GFP that varies its brightness in response to intracellular pH changes.

## Introduction

Genetically encoded fluorescent sensors often suffer from extensive background fluorescence (Lin and Schnitzer, 2016; Bayguinov et al., 2017; Song et al., 2017; Nakajima and Baker, 2018). Confining the expression to naturally sparse cell types such as parvalbumin positive or somatostatin positive interneurons can alleviate the dense expression issue (Lou et al., 2016; Marshall et al., 2016). Co-injecting a Cre recombinase virus together with a floxed virus that contains a gene of interest can provide a sparse expression pattern but this necessitates an optimization step for each virus sample (Xu et al., 2012). Using a destabilized Cre recombinase was reported to induce sparse labeling in cortical layers 2/3 (Sando et al., 2013; Harris et al., 2014; Madisen et al., 2015). Utilizing this method to achieve a Förster resonance energy transfer (FRET) type genetically encoded voltage (GEVI) expression involved the generation of triple transgenic mice to acquire the desired level of sparseness in cortical pyramidal cells (Song et al., 2017; Quicke et al., 2019). However, sparse labeling is a stochastic approach that is difficult to control. Approaches that empower the experimenter to define the circuits that optically respond would be a welcome addition to the imaging toolbox.

Optically activating fluorophores in a region of interest may address this issue (Berlin et al., 2015). Photoactivation of fluorescent proteins enables optical labeling of a defined region (Lukyanov et al., 2005). As such, the rationally designed, photoactivatable GFP (PA-GFP) has been a useful tool since its development (Patterson and Lippincott-Schwartz, 2002; Betzig et al., 2006). This FP exhibited fluorescence when excited at 470 nm only after photoactivation at 413 nm. The photoactivatable genetically-encoded calcium indicator (sPA-GCaMP6f) reported by Berlin et al. (2015) demonstrated the usefulness of it in studying calcium dynamics of neuronal cells. Besides from the photoactivatable version, two green to red photoconvertible genetically encoded calcium indicators were previously reported (Hoi et al., 2013; Fosque et al., 2015). However, since the observation of intracellular calcium transients is an indirect measure of neuronal depolarization (Lin and Schnitzer, 2016; Storace et al., 2016), having a photoactivatable GEVI (PA-GEVI) would be a valuable addition. A photoconvertible voltage indicator was previously reported (Abdelfattah et al., 2016). This GEVI exhibited a change in emission from green to red upon a 400 nm wavelength excitation. The only photoactivatable GEVI reported to date is a microbial rhodopsin voltage indicator with voltage-dependent near-infrared fluorescence requiring intense and simultaneous dual excitation for the photoactivation enhancement and epifluorescence imaging (Adam et al., 2019).

A genetically encoded pH indicator (GEPI) is useful in studying network activity of the brain slice as well as synaptic transmission at the cellular level (Lin and Schnitzer, 2016). Previously, Miesenböck et al. (1998) and Sankaranarayanan et al. (2000) imaged vesicle release dependent pH changes by using pH-sensitive GFPs. Additionally, Raimondo et al. (2012, 2013, 2016) used a ratiometric pH-sensitive GFP to optically study activity-dependent acidification of both neurons and astrocytes in hippocampal slices (Bizzarri et al., 2006; Raimondo et al., 2012, 2016). Therefore, the development of an optically activatable pH indicator would be a useful asset to the optical imaging toolset.

In this article, we describe the design strategies and experimental results of developing photoactivatable voltage and pH indicators. Based on the rationales used for the development of the original PA-GFP and the photoactivatable GCaMP, three candidate PA-GEVIs were developed. These candidates include a circularly permuted FP, a FRET pair, and a pH-sensitive fluorescent protein. One of the candidates, PA-Bongwoori-R3 demonstrated a voltage-dependent fluorescence change only after photoactivation. The fluorescent protein in PA-Bongwoori-R3 is also pH-sensitive. Since the optical bio-sensor field lacks a photoactivatable, genetically encoded pH indicator (Lukyanov et al., 2005; Lippincott-Schwartz and Patterson, 2009; Chudakov et al., 2010), a voltage insensitive variant of the pH-sensitive GFP, PA-ecliptic pHluorin was developed. When this cytoplasmic photoactivatable pH indicator was expressed in mammalian cells, it had changes in fluorescence intensity for varying intracellular pH values that were difficult to measure before photoactivation.

## Materials and Methods

### Gene Constructs Design and Cloning

Two versions of photoactivatable ASAP1 (St-Pierre et al., 2014) were prepared. ASAP1 with three photoactivatable mutations (ASAP1-PATM; Berlin et al., 2015) was generated by two separate gene cloning steps. L163F and T164S mutations (correspond to L64F and T65S mutations in the original PA-GFP) were first introduced by polymerase chain reaction (PCR) using SM067A and SM067B primers. Then the T59H mutation that corresponds to the T203H mutation in PA-GFP construct was substituted in by PCR using SM068A and SM068B primers. A PCR with SM070 and SM071Xho1 primers was conducted to cut and paste the whole ASAP1-PATM insert into pcDNA3.1(+) backbone vector. A second type of photoactivatable ASAP1 was generated by switching the OPT (optimum) variant of circularly permuted superfolder GFP (cpsfGFP-OPT) in ASAP1 into a photoactivatable circularly permuted GFP from the short superfolder photoactivatable GCaMP6f (ssPA-GCaMP6f; Berlin et al., 2015). The synthesized gene fragment had Nhe1 and Xho1 restriction sites to facilitate cloning (Integrated DNA technologies, Coralville, IA, USA; Supplementary Data S1). Restriction digest was carried out with the two enzymes to cut and paste the synthesized gene fragment and it was then ligated into the pcDNA3.1(+) backbone vector to acquire ASAP1-ssPA version.

A FRET version PA-GEVI was prepared by mutating Nabi 2.242 developed by Sung et al. (2015). The original Nabi 2.242 has Clover and mRuby2 as a FRET pair. To replace Clover with PA-GFP, a 1,279 base pair long gene fragment that includes a Nhe1 site, PA-GFP, and an Apa1 site was synthesized (Integrated DNA Technologies, Coralville, IA, USA; Supplementary Data S1) and cloned into the Nabi 2.242 vector (Sung et al., 2015). Another set of PCR was conducted to produce PA-Nabi2.242 where SM100A and SM100B primers were used to introduce PA-Nabi2.242 into the pcDNA3.1(+) backbone vector.

Photoactivatable Bongwoori-R3 (PA-Bongwoori-R3) was prepared by replacing Bongwoori-R3’s (Lee et al., 2017) fluorophore with a mutated ecliptic pHluorin (Supplementary Data S1, Miesenböck et al., 1998). T203H and A227D mutations were introduced in the synthesized gene fragment to confer photoactivatability and voltage-sensitivity to the original ecliptic pHluorin. BamH1 and Xho1 restriction sites were used for cloning into the Bongwoori-R3 gene construct. A voltage-sensitive but non-photoactivatable version (ecliptic-Bongwoori-R3_A227D) and a photoactivatable but non-voltage-sensitive version (PA-Bongwoori-R3_T203H_D227A) were generated by PCR with SM105A and SM105B, and SM106A and SM106B primers, respectively. A cytoplasmic version of photoactivatable GFP (pPA-GFP-N1) was purchased from Addgene, USA (#11909).

A cytoplasmic version of photoactivatable ecliptic pHluorin was generated by a simple one-step PCR with SM103 and SM013 to acquire the ecliptic pHluorin part from PA-Bongwoori-R3_T203H_D227A. The polymerized insert was cut and ligated back into pcDNA3.1(+) backbone vector resulting in photoactivatable ecliptic pHluorin D227A (PA-ecliptic pHluorin).

All primers are listed in Supplementary Table S1. The sequences of newly generated gene constructs were verified commercially (Cosmogenetech, South Korea).

### Cell Culture and Transfection

Human Embryonic Kidney 293 (HEK 293) cells were cultured and transfected following the methods described in Lee et al. (2017) with a few modifications. The cells were maintained in Dulbecco’s Modified Eagle Medium (DMEM; Gibco, USA) with 10% (v/v) Fetal Bovine Serum (Gibco, USA) and kept in a CO_2_ incubator (MCO-20AIC, Sanyo, Japan) at 37°C and 5% CO_2_ level throughout the culture. For a transient transfection with a plasmid DNA construct, the HEK 293 cells were detached by using 0.25% trypsin-EDTA solution (Gibco, USA) and seeded onto poly-D-lysine (Sigma-Aldrich, USA) coated coverslips (10 mm diameter and 0.08–0.13 mm thickness, Ted Pella, USA). For transfection, 1 μL of a lipofection reagent (Lipofectamine 2000, Life Technologies, USA) was pre-mixed with 100 ng of the plasmid DNA molecules for each of the coverslip and incubated in the CO_2_ incubator overnight.

### Photoactivation and Imaging

A 385 nm light-emitting diode [bandwidth (FWHM): 10 nm] placed in a 4-wavelength LED housing (LED4D242, Thorlabs, USA) was used for photoactivation of all photoactivatable variants. To find a photoactivatable optical sensor expressing cell and for photoactivation with the 385 nm LED, a filter cube consisting of a 385/23 nm excitation filter (FF01-386/23-25, Semrock, USA), a 495 nm dichroic mirror (FF495-Di03, Semrock, USA) and a 520/35 nm emission filter (FF01-520/35, Semrock, USA) was used. After photoactivation, a 470 nm LED [bandwidth (FWHM): 25 nm] delivered excitation light to green fluorophores in the specimen. A 4-channel LED driver and its software (DC4100, Thorlabs, USA) were used to control the LEDs. Protocols for simultaneous voltage imaging and electrophysiology experiments were as described in Lee et al. (2017). The high-speed CCD camera (Neuro CCD, RedShirtImaging, Decatur, GA, USA) and the camera software NeuroPlex (RedShirtImaging, Decatur, GA, USA) were used to acquire images. For photoactivation at 385 nm and epifluorescence imaging at 470 nm, the LEDs were modulated at their full intensities. The intensities of 385 nm and 470 nm were 2.7 mW/mm^2^ and 5.3 mW/mm^2^, respectively. Before the photoactivation, the 385 nm LED was used at its 10% intensity to locate transfected cells while preventing unintended photoactivation.

To locate a cell expressing the FRET sensors (PA-Nabi 2. 242), a 565 nm LED [bandwidth (FWHM): 104 nm], a 561/14 nm excitation filter (FF01-561/14, Semrock, USA), a 561 nm dichroic mirror (Di02-R561, Semrock, USA) and a 609/54 nm emission filter (FF01-609/54, Semrock, USA) were used. For the imaging of a FRET pair, an image splitter (Optosplit 2, Cairn, UK) was placed between the high-speed CCD camera and the c-mount port of the microscope to divide the CCD sensor into two halves for simultaneous imaging of green (ET 520/40, Chroma, USA) and red (ET 645/75, Chroma, USA) fluorescence. For FRET imaging, the 470 nm LED light was filtered by a 475/23 nm excitation filter (FF01-475/23-25, Semrock, USA). For imaging of Nabi 2.242, a 75 W Xenon arc lamp (Osram, Germany) was used (lamp housing from Cairn, UK).

For pH imaging experiments of PA-GFP and PA—ecliptic pHluorin, and Gramicidin D (Sigma-Aldrich) was applied to transfected cells at 50 μM to perforate their cell membranes for 20 min at 34°C. The Gramicidin was prepared and stored as described previously (Kang and Baker, 2016). After the photoactivation, each bath solution with a different pH was perfused into the patching chamber for at least 20 min to induce pH-dependent fluorescence change of the photoactivatable probe. The strongly buffered bath solution (100 mM NaCl, 3 mM KCl, 0.5 mM MgCl_2_, 1 mM CaCl_2_, 3 mM Glucose and 100 mM HEPES) was prepared following the description in Kang and Baker (Kang and Baker, 2016) and used to change intracellular pH level. A scientific CCD camera (128 × 128 pixels, Neuro CCD, RedShirtImaging, Decatur, GA, USA) was used to measure pH sensitivities of the fluorescent proteins.

### Analyses

As described earlier in Lee et al. (2017), fluorescence traces from selected pixels were derived from NeuroPlex software (RedShirtImaging, Decatur, GA, USA). Calculation of averaged ΔF/F values and statistics of means for comparison were conducted in Origin 9.0 (OriginLab, Northampton, MA, USA) and Microsoft Office—Excel 2016 (Microsoft, USA).

## Results

### Engineering a Circularly Permuted FP Type GEVI

The photoactivatable GFP (PA-GFP) developed by Patterson and Lippincott-Schwartz (2002) had four mutations in comparison to eGFP. The L64F and T65S mutations recovered the 390 nm absorbance peak that originally existed in wild-type GFP (wtGFP; The amino acid residue numbers indicate GFP residues only). The T203H mutation maximized the contrast of the two absorbance bands so the FP could stay dim during 470 nm excitation until it was activated by 390 nm light. The V163 residue was mutated to alanine to improve protein folding at 37°C as an alternative to the F64L mutation in eGFP.

**Figure 1:**
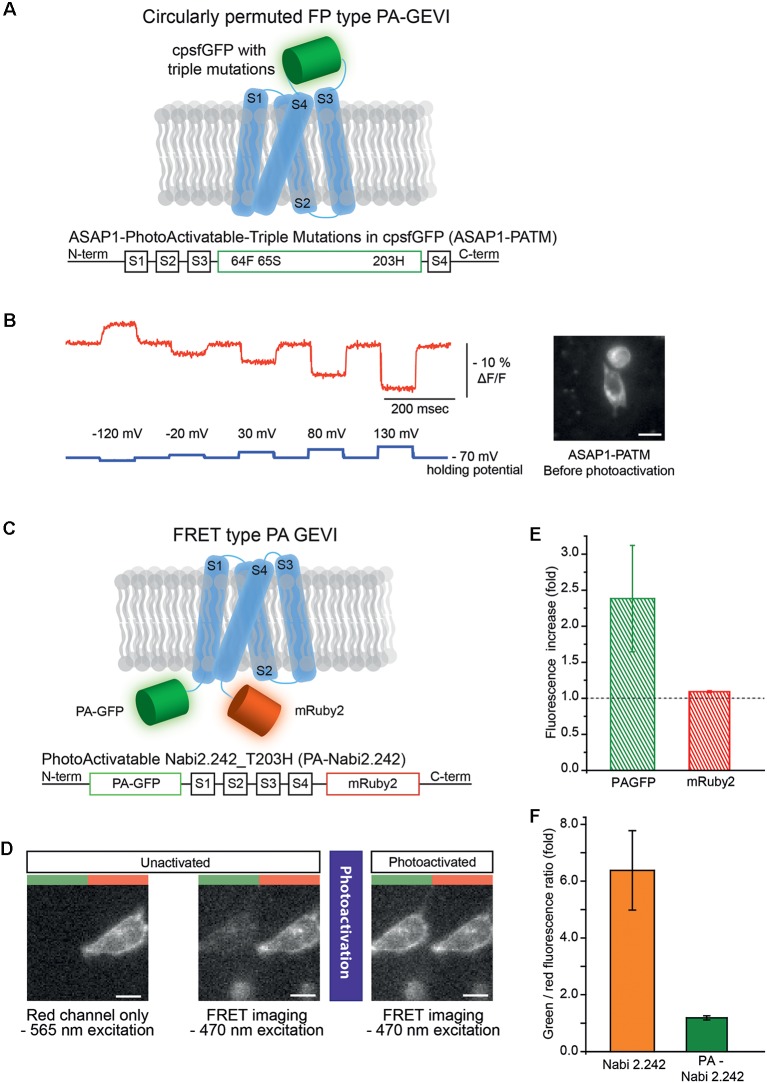
Photoactivatable versions of ASAP1 and Nabi 2.242. **(A)** Schematics of photoactivatable ASAP1-PATM (top) and the gene construct design (bottom). The voltage-sensing domain is depicted in light blue. **(B)** A representative voltage-dependent fluorescence change of the unactivated ASAP1-PATM. The inset on the right shows an HEK 293 cell expressing ASAP1-PATM before photoactivation. **(C)** Schematics of photoactivatable Nabi 2.242 (PA-Nabi 2.242; top) and its gene construct design (bottom). **(D)** Images acquired by a CCD camera before (left/middle) and after photoactivation (right). The left half of each image represents FRET donor (green) channel and the right half is the FRET acceptor (red) channel. **(E)** The increase in fluorescence after photoactivation for PA-GFP and mRuby2. **(F)** The ratio between FRET donor (green) and FRET acceptor (red) fluorescence calculated for the original non-photoactivatable Nabi 2.242 (Clover/mRuby2 brightness ratio) and PA-Nabi 2.242 (photoactivated PA-GFP/mRuby2 brightness ratio), respectively. For the fluorescence imaging of Nabi 2.242, a Xenon arc lamp was used. The photoactivation time for PA-Nabi 2.242 cells varied for 30 s (cell1), 10 s (cell2), and 20 s (cell3). Scale bar is 20 μm.

Two photoactivatable voltage indicator candidates were designed based on the rationale described above (Figures 1A,C). First, a photoactivatable version of ASAP1 (St-Pierre et al., 2014) which is a genetically-encoded voltage indicator with a voltage-sensing domain and a circularly permuted GFP was generated. The three mutations from PA-GFP, F64L, S65T and T203H, were introduced into the GEVI’s FP and named as ASAP1-PATM (Figure 1A, bottom). The V163A mutation that was previously used to improve protein folding was already present in ASAP1. Another photoactivatable variant of ASAP1 was prepared by using a photoactivatable circularly permuted GFP engineered by Berlin et al. (2015) for the development of their photoactivatable calcium indicators. The FP part of ASAP1 was substituted with the short superfolder photoactivatable circularly permuted GFP (sPA-cpGFP). This candidate was named ASAP1-ssPA.

ASAP1-ssPA transfected HEK 293 cells were imaged with both 385 nm and 470 nm excitation light. Very weak green fluorescence was observed indicating poor expression of the probe (Supplementary Figure S1). Conversely, ASAP1 with the three photoactivatable mutations (ASAP1-PATM) expressed well in the plasma membrane (Figure 1B, inset). The photoactivatable ASAP1 showed bright green fluorescence even before the photoactivation with 385 nm light. In its non-photoactivated state, this GEVI responded well to voltage steps (Figure 1B). The response in the non-photoactivated state diminished the value of this probe as a photoactivatable optical sensor.

### Engineering a GEVI With a FRET Pair

A photoactivatable GEVI with a FRET pair was also generated. Nabi 2.242 is a FRET-based GEVI (Sung et al., 2015) that consists of the FPs, Clover, the FRET donor and, mRuby2, the FRET acceptor (Lam et al., 2012). Originally, Nabi 2.242 responds to a depolarizing voltage pulse with a decrease in the FRET donor and an increase in the acceptor fluorescence. Since the photoactivated PA-GFP had excitation and emission spectra similar to Clover (Patterson and Lippincott-Schwartz, 2002; Lam et al., 2012), it was substituted as the FRET donor (Figure 1C).

To verify the expression in HEK 293 cells, mRuby2 was excited with a 565 nm LED which kept the photoactivatable FRET donor inactive (Figure 1D, left). The image from the FRET acceptor channel clearly showed red fluorescence mainly from the plasma membrane of the cell. Subsequent FRET imaging with a 470 nm LED showed weak green and red fluorescence indicating a basal level of FRET before photoactivation (Figure 1D, middle). After photoactivation of the FRET donor, the PA-GFP emission was increased about 2-fold (Figure 1D, right and (Figure 1E) but the FRET acceptor showed only 10% increase in its red fluorescence (Figure 1E). The green to red fluorescence ratios of the original non-photoactivatable Nabi 2.242 (Clover/mRuby2) and PA-Nabi 2.242 (photoactivated PA-GFP/mRuby2) were analyzed to compare the relative brightness of Clover and photoactivated PA-GFP (Figure 1F). This revealed that the photoactivated PA-GFP was 5-fold dimmer than the original FRET donor FP. There was no detectable voltage-dependent optical signal.

### Development of Photoactivatable Bongwoori-R3 With a Mutated Ecliptic pHluorin

**Figure 2:**
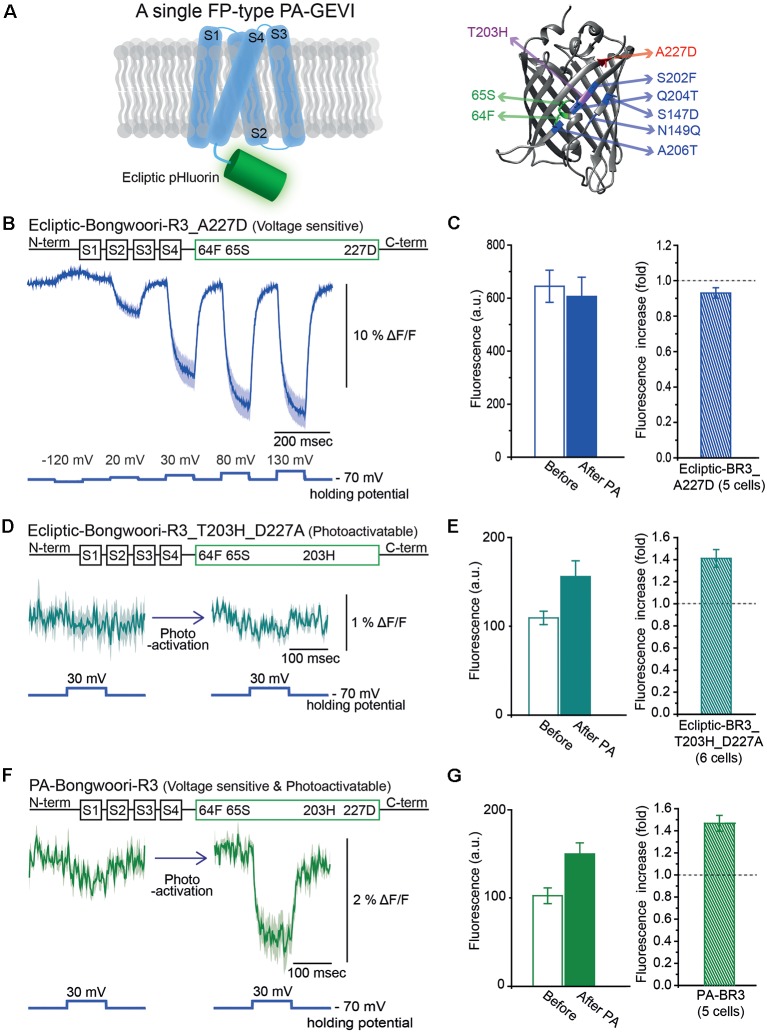
Engineering of Photoactivatable Bongwoori-R3 and verification of its photoactivatability and voltage sensitivity. **(A)** A schematic design of Bongwoori-R3 with an ecliptic pHluorin (left). Key mutations of photoactivatable ecliptic pHluorin compared to wild-type GFP (right). Residues in green (65S and 64F) contribute to the wild-type dual absorbance bands. The T203H mutation is responsible for photoactivatability. The A227D mutation confers voltage-sensitivity. Mutations depicted in blue color (S147D, N149Q, S202F, Q204T and A206T) are characteristic residues for ecliptic pHluorin compared to wtGFP. Protein Data Bank ID: 1GFL. **(B)** Gene construct design of ecliptic-Bongwoori-R3 with the A227D mutation (top) and the voltage-dependent fluorescence change imaged from HEK 293 cells expressing the variant (bottom). Traces from three cells were analyzed. **(C)** Raw fluorescence intensity values imaged before and after photoactivation (left). The ratio of the fluorescence change is on the right. The number of cells analyzed was five. **(D)** Gene construct design (top) and response to a 100 mV voltage pulse (bottom) of ecliptic-Bongwoori-R3_T203H_D227A. **(E)** The raw fluorescence intensities before and after photoactivation (left). The increase ratio after photoactivation (right). Six cells were analyzed. **(F)** PA-Bongwoori-R3 responding to a 100 mV membrane depolarization. **(G)** The fluorescence intensities before and after photoactivation (left). The increase ratio of fluorescence intensity after photoactivation (right). Five PA-Bongwoori-R3 expressing cells were analyzed. The shaded area in the traces and error bars denote standard error of the mean. The protein structure image in **(A)** was from Protein Data Bank ID: 1GFL. Further modifications were made with UCSF Chimera 1.13.1 software.

We also tried to develop a photoactivatable version of the GEVI, Bongwoori-R3 (Lee et al., 2017). Bongwoori-R3 uses the FP, super ecliptic pHluorin, to optically report voltage changes. Super ecliptic pHluorin is a modified version of ecliptic pHluorin with diminished 390 nm excitation (Miesenböck et al., 1998; Sankaranarayanan et al., 2000). We, therefore, created a photoactivatable probe with the ecliptic version of the FP that can be excited with both 390 nm and 470 nm light (Figure 2A). The ecliptic FP version of ArcLight has been shown to be voltage-sensitive (Jin et al., 2012; Treger et al., 2015; Platisa et al., 2017). The ecliptic Bongwoori-R3 construct exhibited a voltage-dependent fluorescence change that was smaller and slower than that of the original Bongwoori-R3 (Lee et al., 2017; Figure 2B). This variant did not show an increase in fluorescence after photoactivation (Figure 2C). The construct consisting of ecliptic-Bongwoori-R3 with the T203H photoactivatable mutation but lacking the A227D mutation that improves the voltage-dependent optical signal was prepared. This construct increased fluorescence upon photoactivation but did not yield a voltage-sensitive optical signal (Figure 2D). The photoactivation with 385 nm light improved its brightness by 1.4 ± 0.1 fold (Figure 2E).

Subsequently, both the A227D and T203H mutations were introduced into the ecliptic pHluorin version of Bongwoori-R3 to improve voltage sensitivity and confer photoactivatability. This variant of Bongwoori-R3 expressed well in HEK 293 cells. A photoactivation at 385 nm wavelength increased the green fluorescence imaged with 470 nm excitation light about 1.5 ± 0.1-fold (Figure 2G). To examine its voltage-sensitivity, a 100 mV voltage change was induced before and after the photoactivation while imaging at a frame rate of 1,000 Hz. Before photoactivation, only a slight voltage-dependent fluorescence change was observed (Figure 2F, left). After photoactivation, a 2% Δ F/F was observed during the 100 mV depolarization step. While this signal size is much reduced compared to the original Bongwoori-R3 probe, the fact that the voltage-dependent optical signal can only be detected after photoactivation suggests that the probe may be useful *in vivo* and merits further development. We named this variant PA-Bongwoori-R3.

### Development of Photoactivatable Ecliptic pHluorin and Its pH Sensitivity

**Figure 3:**
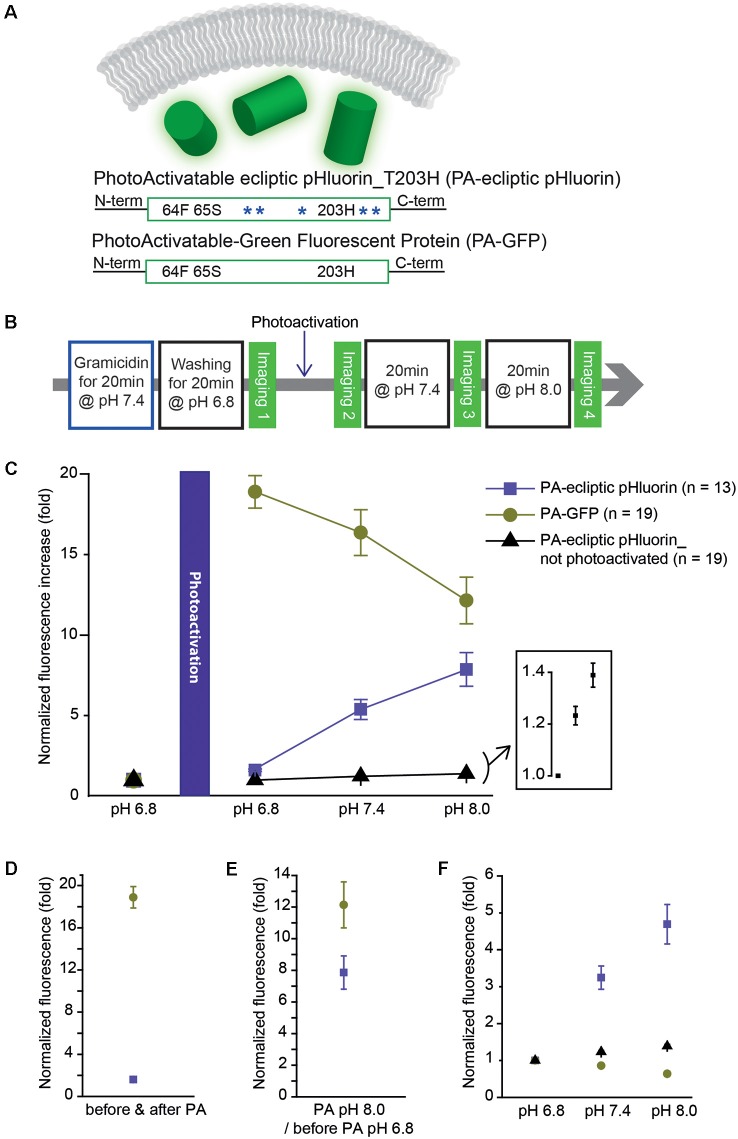
A photoactivatable optical pH sensor (PA-ecliptic pHluorin) and its pH sensitivity in HEK 293 cells in comparison to PA-GFP. **(A)** Schematics of a cytoplasmic optical pH sensor (top), the photoactivatable ecliptic pHluorin with T203H mutation (middle) and PA-GFP (bottom). The blue asterisks depict pH-sensitive mutations in ecliptic pHluorin. **(B)** Experimental procedure for the photoactivatability and pH sensitivity tests. **(C)** Fluorescence intensities measured at each pH level normalized to the fluorescence read-out at pH 6.8 before photoactivation. For the non-photoactivated PA-ecliptic pHluorin experiment (black dots), the photoactivation step shown in **(B)** was omitted. The inset shows PA-ecliptic pHluorin’s sensitivity at pH 6.8, 7.4, and 8.0 from the no photoactivation experiment. **(D)** Comparison of photoactivatability of PA-GFP (dark green dots) and PA-ecliptic pHluorin (violet dots) at pH 6.8 (normalized to non-photoactivated states at pH 6.8). **(E)** Fluorescence intensities of PA-GFP (dark green dots) and PA-ecliptic pHluorin (violet dots) at photoactivated pH 8.0 normalized to non-photoactivated states at pH 6.8. **(F)** Fluorescence intensities of PA-GFP (dark green dots) and PA-ecliptic pHluorin (violet dots: photoactivated/black dots: non-photoactivated) normalized to their values at pH 6.8 after photoactivation to only show their pH sensitivities. Error bars are standard error of the mean (SEM). The number of cells analyzed were 13 cells (PA-ecliptic pHluorin), 19 cells (PA-GFP) and 19 cells (PA-ecliptic pHluorin_non-photoactivated).

The photoactivatable version of Bongwoori-R3 used a variant of a pH-sensitive GFP, ecliptic pHluorin, which is known to get brighter as the pH increases. A photoactivatable GEPI candidate was created by removing the *Ciona Intestinalis* voltage-sensing domain from ecliptic-Bongwoori-R3_T203H_D227A. Since it was unnecessary for the probe to sense a voltage change, the 227D mutation in the FP was also removed (Figure 3A). The cytoplasmic version of ecliptic pHluorin with T203H (PA-ecliptic pHluorin) and PA-GFP were expressed in HEK 293 cells. The cells were first incubated with an ionophore that makes the cell membrane permeable to external monovalent cations (Gramacidin, Sigma-Aldrich, USA). Next, the cells were washed with a pH 6.8 bath solution with a high buffering capacity (100 mM HEPES) to efficiently change the intracellular pH (Figure 3B). Subsequent photoactivation of both PA-ecliptic pHluorin and PA-GFP at the same pH level increased the fluorescence intensity by 1.5 and 18-fold, respectively (Figures 3C,D). As the pH of the bath solution was changed from 6.8 to 7.4, PA-ecliptic pHluorin showed an increase in its fluorescence level as expected from ecliptic pHluorin’s pH sensitivity (Miesenböck et al., 1998). At pH 8.0, PA-ecliptic pHluorin exhibited a further increase in green emission that was 7.9 ± 1.0 fold brighter compared to its non-activated state at pH 6.8 (Figure 3E). The non-pH sensitive FP, PA-GFP, only showed decreased fluorescence levels as the bath solution was changed to basic pH. Photoactivation did not confer pH sensitivity. As a control experiment, non-photoactivated PA-ecliptic pHluorin was also tested at different pH levels. Although the fluorescence from these cells remained dim, the FP was slightly sensitive to pH changes (Figure 3C, inset). Figure 3F summarizes pH sensitivities from the three experiments.

## Discussion

The addition of PA-GFP to the palette of fluorescent proteins led to the development of photoactivated localization microscopy (PALM) that realized single molecule imaging below the diffraction limit in a biological sample (Patterson and Lippincott-Schwartz, 2002; Betzig et al., 2006; Lippincott-Schwartz and Patterson, 2009). The wtGFP has some intrinsic degree of photoactivation due to its characteristic dual excitation bands at 397 nm and 475 nm. Before photoactivation, its chromophore stays in the protonated (neutral) state. Illumination with near 400 nm light is thought to shift the neutral state into the anionic state by deprotonating the chromophore. In this activated state, the excitation at 470 nm becomes dominant. PA-GFP has the T203H mutation that improves the 470 nm excitation upon photoactivation with 400 nm light from 3-fold (wtGFP) to 100-fold (PA-GFP) when measured using purified protein preparations (Patterson and Lippincott-Schwartz, 2002). In order to utilize the advantage of photoactivatable fluorescent proteins for the efficient and controllable fluorescence imaging of voltage and pH, several photoactivatable GEVI candidates and one photoactivatable GEPI were rationally designed and generated. Except for ASAP1-ssPA, all candidates expressed well in HEK 293 cells and showed an increase in fluorescence upon photoactivation. The ASAP1-PATM was bright enough to resolve induced voltage pulses even before photoactivation. However, it would be more useful if the increase in fluorescence level after photoactivation is large enough to make the activated cells easily distinguishable from non-activated cells.

The 385 nm LED light used to photoactivate all the photoactivatable variants in this work was measured to be 2.7 mW/mm^2^ in intensity. As this light intensity was both low and slightly off centered from the typical 400–405 nm light used for illuminating a photoactivatable GFP, the increase after photoactivation may improve if a stronger 400–405 nm light source is used.

The FRET version of the GEVI, PA-Nabi 2.242 was prepared by replacing the bright green FP, Clover, by PA-GFP. This construct failed to show a voltage-dependent FRET signal. Several properties affect FRET efficiency. The distance between the chromophores of donor and acceptor, the spectral overlap between the donor’s emission and the acceptor’s excitation bands, and the relative orientation of the donor and acceptor dipoles all contribute to the FRET efficiency (Tsien, 1998; Campbell, 2009). Both PA-GFP and Clover are variants of GFP and the construction of PA-Nabi 2.242 was a simple substitution of PA-GFP for Clover. The emission maxima of the two FPs (photoactivated PA-GFP and Clover) are 517 nm and 515 nm, respectively. A comparison of the emission spectra suggests the overlap with the mRuby2’s excitation spectrum was similar for both FRET donors. The quantum yields of photoactivated PA-GFP and Clover are 0.79 and 0.76, respectively (Lukyanov et al., 2005; Lam et al., 2012). However, the extinction coefficients for photoactivated PA-GFP and Clover were measured to be 17,400 and 111,000 M^−1^ cm^−1^ (Patterson and Lippincott-Schwartz, 2002; Lam et al., 2012). As the intrinsic brightness of a fluorophore is determined by extinction coefficient multiplied by quantum yield, Clover is far brighter than the photoactivated PA-GFP. The brightness of photoactivated PA-GFP was measured to be 0.42-fold of eGFP (Lukyanov et al., 2005). Clover is about 2.5-fold brighter than eGFP (Lam et al., 2012). A rough extrapolation of the two values would suggest that the photoactivated PA-GFP is about 5–6-fold dimmer than Clover. A similar difference was seen in our result as well (Figure 1F). Although PA-Nabi 2.242 was not promising, having a red-shifted FP as both a FRET acceptor and a guide to locate nicely transfected cells without accidentaly activating photoactivatable GFP molecules merits continued exploration.

Photoactivatable Bongwoori-R3 and its derivatives nicely demonstrated the functions of the voltage sensing mutation (A227D) and the photoactivatable mutation (T203H). The voltage-sensitive but non-photoactivatable version, ecliptic-Bongwoori-R3_A227D, had a small voltage-dependent fluorescence change without photoactivation. However, the ΔF/F for a 100 mV depolarization of the plasma membrane was decreased to less than 50% of the original Bongwoori-R3. Jin et al. (2012) also tested both ecliptic pHluorin and super ecliptic pHluorin containing the A227D mutation. According to their report, both variants showed about 18% ΔF/F for a 100 mV depolarization. Further linker length optimization for the super ecliptic pHluorin A227D version resulted in the development of ArcLight. Since it is well-known that both linker length and composition affect the voltage-dependent fluorescence signal (Jung et al., 2015; Piao et al., 2015; Lee et al., 2017; Yi et al., 2018), the same may improve PA-Bongwoor-R3 variants’ optical signals. Combining the two distinct mutations successfully accomplished a photoactivatable GEVI. With the optical setup that was used in this work, PA-Bongwoori-R3 expressing cells responded to a voltage pulse only after they were photoactivated. As these cells stay in a dim state before the activation, even the modest ΔF/F change could be useful (Figure 2F).

The intensity of PA-Bongwoori-R3 after photoactivation showed less than a 2-fold increase. This was about 50-fold weaker than that previously reported for PA-GFP (Patterson and Lippincott-Schwartz, 2002). Although the 100-fold increase from the original report was measured from purified proteins embedded in a polyacrylamide gel, PA-Bongwoori-R3’s photoactivation increase was modest. This may suggest that the mutations in the photoactivatable ecliptic pHluorin A227D hindered proper photoactivation. Another possibility could be the weak 385 nm LED light used to photoactivate the molecules. The small 2% ΔF/F per 100 mV of PA-Bongwoori-R3 could also be improved through the optimization of linker length.

The regulation of intracellular proton concentration is involved in synaptic vesicle release and network excitability (Sankaranarayanan et al., 2000; Raimondo et al., 2012, 2013, 2016; Lin and Schnitzer, 2016). Optical measurement of intracellular pH changes is important since other means to measure pH such as microelectrodes are limited due to their size (Raimondo et al., 2013). The presynaptic terminal of a neuron, the pH level in the synaptic vesicle lumen is maintained near pH 5.5 and increases to pH 7.3 upon the vesicle opening into the synaptic cleft (Anderson and Orci, 1988; Nelson, 1992; Lin and Schnitzer, 2016). Ecliptic pHluorin is only weakly fluorescent at pH 5.5 and becomes brighter at pH 7.3 (Miesenböck et al., 1998). The pH-sensitivity of PA-ecliptic pHluorin should be useful for studying pH changes in the cytoplasm (Figure 3C). Non-photoactivated PA-ecliptic pHluorin was still pH-sensitive but its brightness at pH 8.0 increased by about 1.5-fold compared to the photoactivated state which had an 8-fold increase in fluorescence.

The key finding of this report is that the voltage-sensing mutation, A227D, and the photoactivatable mutation, T203H, were applicable to the development of photoactivatable voltage and pH sensors. Further optimization of photoactivation for both PA-Bongwoori-R3 and PA-ecliptic pHluorin may enable the optical resolution of both high speed and low speed *in vivo* neuronal activity.

## Data Availability Statement

The datasets generated for this study are available on request to the corresponding author.

## Author Contributions

SL designed experiments, performed experiments, analyzed data, created figures and wrote the manuscript. Y-KS analyzed data and co-wrote the manuscript. BB designed experiments, analyzed data, and co-wrote the manuscript.

## Conflict of Interest

The authors declare that the research was conducted in the absence of any commercial or financial relationships that could be construed as a potential conflict of interest.
