# Supplementary material for: Engineering Photoactivatability in Genetically Encoded Voltage and pH Indicators
Source: Front Cell Neurosci. 2019 Oct 29;13:482. doi: 10.3389/fncel.2019.00482 (PMC6828978; doi:10.3389/fncel.2019.00482)
Supplement: Supplementary file 1 [file Table_1.docx]

Supplementary Material

# Supplementary Data 1. Synthesized gene constructs

A. ASAP1-ssPA-cpGFP version_Nhe1Xho1
TTT GCT AGC CGC CAC CAT GGA GAC GAC TGT GAG GTA TGA ACA GGG GTC AGA GCT CAC TAA AAC TTC GAG CTC TCC AAC AGC AGA TGA GCC CAC GAT AAA GAT TGA TGA TGG TCG TGA TGA GGG TAA TGA ACA AGA CAG CTG TTC CAA TAC CAT TAG GAG AAA AAT TTC CCC GTT TGT GAT GTC ATT TGG ATT CAG AGT ATT TGG AGT TGT GCT TAT CAT TGT AGA CAT CAT AGT GGT GAT TGT GGA TCT GGC CAT CAG TGA GAA GAA AAG AGG CAT TAG AGA GAT TCT TGA AGG TGT TTC CCT GGC TAT AGC ACT CTT CTT CCT TGT TGA TGT TCT CAT GAG AGT GTT TGT TGA AGG CTT CAA GAA CTA TTT CCG GTC CAA ACT GAA TAC TTT GGA TGC AGT CAT AGT AGT GGG CAC TCT GCT AAT TAA TAT GAC CTA CTC CTT CTC TGA CCT TGC TGC CAA CGT CTA TAT CAC CGC CGA CAA GCA GAA GAA CGG CAT CAA GGC GAA CTT CAA GAT CCG CCA CAA CGT GGA GGA CGG CAG CGT GCA GCT CGC CGA CCA CTA CCA GCA GAA CAC CCC CAT CGG CGA CGG CCC CGT GCT GCT GCC CGA CAA CCA CTA CCT GAG CCA TCA GTC CGT GCT TTC GAA AGA CCC CAA CGA GAA GCG CGA TCA CAT GGT CCT GCT GGA GTT CGT GAC CGC CGC CGG GAT CAC TCT CGG CAT GGA CGA GCT GTA CAA GGG CGG TAC CGG AGG GAG CAT GGT GAG CAA GGG CGA GGA GCT GTT CAC CGG GGT GGT GCC CAT CCT GGT CGA GCT GGA CGG CGA CGT AAA CGG CCA CAA GTT CAG CGT GAG GGG CGA GGG TGA GGG CGA TGC CAC CAA CGG CAA GCT GAC CCT GAA GTT CAT CTG CAC CAC CGG CAA GCT GCC CGT GCC CTG GCC CAC CCT CGT GAC CAC CTT CAG CTA CGG CGT GCA GTG CTT CAG CCG CTA CCC CGA CCA CAT GAA GCA GCA CGA CTT CTT CAA GTC CGC CAT GCC CGA AGG CTA CGT CCA GGA GCG CAC CAT CTC CTT CAA GGA CGA CGG CAC CTA CAA GAC CCG CGC CGA GGT GAA GTT CGA GGG CGA CAC CCT GGT GAA CCG CAT CGA GCT GAA GGG CAT CGA CTT CAA GGA GGA CGG CAA CAT CCT GGG GCA CAA GCT GGA GTA CAA CGA TCA GAT GCC GCA GAT GGT TAC TCT TCT TCG AGT TCT GAG AAT TGT TAT CTT AAT AAG AAT ATT TCG CCT GGC TTC ACA GAA GAA ACA ACT TGA AGT GGT AAC CTA ATG AGC TCG AGT CTA GAG GGC CCG TTT

B. PA-Nabi 2.242_v02
CCC AAG CTG GCT AGC GTT TAT GGA GGG ATT CGA CGG TTC AGA TTT TAG TCC TCC AGC TGA TTT AGT TGG CGT TGA CGG TGC AGT CAT GCG GAA CGT CGT TGA CGT CAC GAT AAA TGG TGA CGT CAC TGC TCC GCC GAA AGC TGC GCC AAG AAA AAG TGA ATC GGT AAA GAA AGT TCA TTG GAA TGA TGT AGA CCA AGG ACC GAG TGA AAA ACC AGA GAC AAG ACA GGA GGA ACG AAT AGA TAT ACC CGA GAT TTC AGG TCT ATG GTG GGG CGA GAA TGA ACA TGG AGT GGA CGA TGG GAG AAT GAT GGT GAG CAA GGG CGA GGA GCT GTT CAC CGG GGT GGT GCC CAT CCT GGT CGA GCT GGA CGG CGA CGT AAA CGG CCA CAA GTT CAG CGT GTC CGG CGA GGG CGA GGG CGA TGC CAC CTA CGG CAA GCT GAC CCT GAA GTT CAT CTG CAC CAC CGG CAA GCT GCC CGT GCC CTG GCC CAC CCT CGT GAC CAC CTT CAG CTA CGG CGT GCA GTG CTT CAG CCG CTA CCC CGA CCA CAT GAA GCA GCA CGA CTT CTT CAA GTC CGC CAT GCC CGA AGG CTA CGT CCA GGA GCG CAC CAT CTT CTT CAA GGA CGA CGG CAA CTA CAA GAC CCG CGC CGA GGT GAA GTT CGA GGG CGA CAC CCT GGT GAA CCG CAT CGA GCT GAA GGG CAT CGA CTT CAA GGA GGA CGG CAA CAT CCT GGG GCA CAA GCT GGA GTA CAA CTA CAA CAG CCA CAA CGT CTA TAT CAT GGC CGA CAA GCA GAA GAA CGG CAT CAA GGC CAA CTT CAA GAT CCG CCA CAA CAT CGA GGA CGG CAG CGT GCA GCT CGC CGA CCA CTA CCA GCA GAA CAC CCC CAT CGG CGA CGG CCC CGT GCT GCT GCC CGA CAA CCA CTA CCT GAG CCA CCA GTC CAA GCT GAG CAA AGA CCC CAA CGA GAA GCG CGA TCA CAT GGT CCT GCT GGA GTT CGT GAC CGC CGC CGG GAT CAC TCT CGG CAT GGA CGA GCT GTA CAA GGA GAT ACC TAC TAC TGG TGT AGG TCG CGT CCA GTT TCG TGT CCG AGC AGT GAT TGA TCA TCT AGG GAT GCG AGT CTT TGG AGT CTT CCT AAT TTT CTT GGA CAT CAT CCT CAT GAT CAT TGA TCT CAG TCT TCC AGG AAA AAG TGA ATC TTC ACA ATC CTT TTA TGA CGG GAT GGC TTT GGC TCT TTC TTG TTA TTT CAT GCT GGA TTT AGG ATT AAG GAT ATT TGC CTA CGG GCC CAA GAA TTT CTT CAC CAA CCC C

C. PA-Bongwoori-R3
CAA GGG GAT CCC ATG AGT AAA GGA GAA GAA CTT TTC ACT GGA GTT GTC CCA ATT CTT GTT GAA TTA GAT GGT GAT GTT AAT GGG CAC AAA TTT TCT GTC AGT GGA GAG GGT GAA GGT GAT GCA ACA TAC GGA AAA CTT ACC CTT AAA TTT ATT TGC ACT ACT GGA AAA CTA CCT GTT CCA TGG CCA ACA CTT GTC ACT ACT TTC TCT TAT GGT GTT CAA TGC TTT TCA AGA TAC CCA GAT CAT ATG AAA CGG CAT GAC TTT TTC AAG AGT GCC ATG CCC GAA GGT TAT GTA CAG GAA AGA ACT ATA TTT TTC AAA GAT GAC GGG AAC TAC AAG ACA CGT GCT GAA GTC AAG TTT GAA GGT GAT ACC CTT GTT AAT AGA ATC GAG TTA AAA GGT ATT GAT TTT AAA GAA GAT GGA AAC ATT CTT GGA CAC AAA TTG GAA TAC AAC TAT AAC GAT CAC CAG GTG TAC ATC ATG GCA GAC AAA CAA AAG AAT GGA ATC AAA GCT AAC TTC AAA ATT AGA CAC AAC ATT GAA GAT GGA GGC GTT CAA CTA GCA GAC CAT TAT CAA CAA AAT ACT CCA ATT GGC GAT GGG CCC GTC CTT TTA CCA GAC AAC CAT TAC CTG TTT CAC ACT TCT ACT CTT TCG AAA GAT CCC AAC GAA AAG AGA GAC CAC ATG GTC CTT CTT GAG TTT GTA ACA GCT GAT GGG ATT ACA CAT GGC ATG GAT GAA CTA TAC AAA TAA TCT AGA ACT CGA GTC T

| Primer | Sequence | Construct |
| --- | --- | --- |
| SM103 | AAGCTGGCTAGCATGAGTAAAGGAGAAGAACTTTTCACTGGAG | To make cytoplasmic PA-ecliptic pHluorin (from ecliptic-Bongwoori-R3_T203H) |
| SM105A | AACCATTACCTGTTTacaACTTCTACTCTTTCGAAAGATCCCAACG | H203T for Photoactivatable _Bongwoori-R3 |
| SM105B | CGAAAGAGTAGAAGTtgtAAACAGGTAATGGTTGTCTGGTAAAAGGAC |  |
| SM106A | TTCTTGAGTTTGTAACAGCTgccGGGATTACACATGGCATGGATGAAC | D227A for Photoactivatable _Bongwoori-R3 |
| SM106B | ATGCCATGTGTAATCCCggcAGCTGTTACAAACTCAAGAAGGACCATG |  |
| SM107 | ttaatgcgccgctacagggcgcgtgggg | Reverse primer for cloning of T203 and A227 positions |
| SM070 | CCACTGAGATCTGCATGAGCTAGCCGCCACCATGG | 5' forward primer for ASAP1 |
| SM071 | CCACCACACTGGACTAGTGGATCCGAGCTCGGTACCAAGC | 3' reverse primer for ASAP1 |
| SM072R1 | GAG TTC CCA TGT ACG GAT TGC CTT CTC C | Two sequencing primers (reverse) for Nabi 2.242 construct |
| SM072R2 | CCT CAT TTT ATT AGG AAA GGA CAG TG |  |
| SM100A | GGAGACCCAAGCTGGCTAGCGTTTatggaggg | Nhe1 site including primer to PCR pUB-pa-Nabi2.242_v02 and paste it into pcDNA3.1+ |
| SM100B | aaaactcgagTCGAGGGCGCGCCTaTTActtgtacagctcg | Xho1 site including primer to PCR pUB-pa-Nabi2.242_v02 and paste it into pcDNA3.1+ |
| SM071XHO1 | TAGACTCGAGTTCTAGATCATTAGGTTACCACTTCAAGTTGTTTC | to insert the PCR product of ASAP1-PATM into pcDNA3.1 |

**Supplementary Table 1. Primer sequences used for gene cloning.**

**
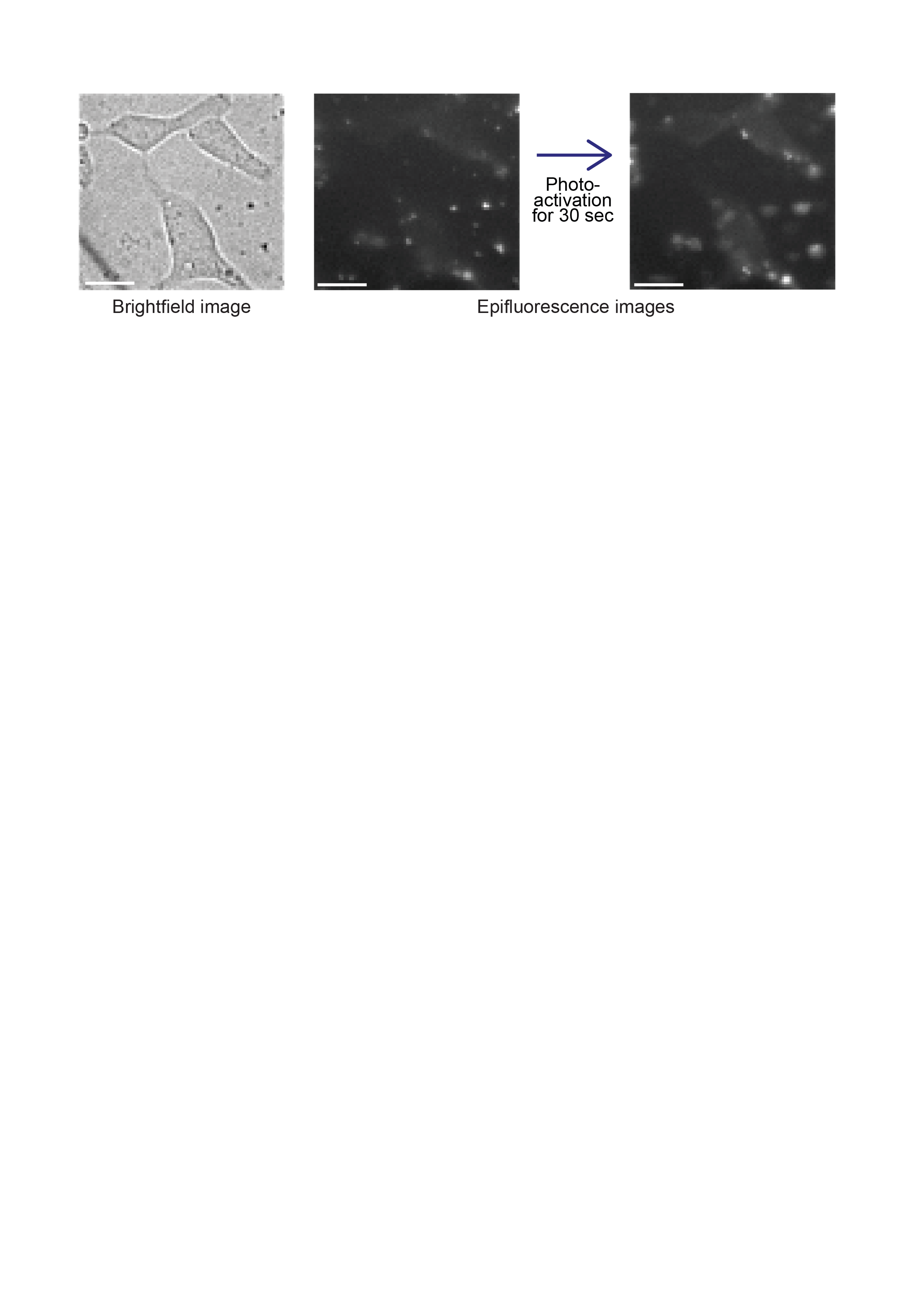
Supplementary Figure 1. ASAP1-ssPA transfected HEK 293 cells showing weak expression.** Note that the cells are showing poor expression even after photoactivation. The epifluorescence images were taken at 40 frames per second. Scale bars are 20 µm.
